# First report of the exotic blue land planarian, *Caenoplana coerulea* (Platyhelminthes, Geoplanidae), on Menorca (Balearic Islands, Spain)

**DOI:** 10.3897/zookeys.199.3215

**Published:** 2012-06-04

**Authors:** Karin Breugelmans, Josep Quintana Cardona, Tom Artois, Kurt Jordaens, Thierry Backeljau

**Affiliations:** 1 Royal Belgian Institute of Natural Sciences, Vautierstraat 29, B-1000 Brussels, Belgium; 2 Institut Catala de Paleontologia Miquel Crusafont, Universitat Autònoma de Barcelona, edifici ICP Campus de la UAB, s/n 08193 Cerdanyola del Vallés, Barcelona, Spain; 3 Research Group Zoology: Biodiversity & Toxicology, Centre for Environmental Sciences, Hasselt University, Campus Diepenbeek, Agoralaan Building D, B-3590 Diepenbeek, Belgium; 4 Joint Experimental Molecular Unit, Royal Museum for Central Africa, Leuvensesteenweg 13, B-3080 Tervuren, Belgium; 5 Evolutionary Ecology Group, University of Antwerp, Groenenborgerlaan 171, B-2020 Antwerp, Belgium

**Keywords:** Terrestrial flatworm, 18S rDNA, COI, introduction, molecular identification, Balearic Islands, Spain, Europe

## Abstract

In April 2009 two specimens of a terrestrial flatworm were collected from under a rock in an orchard at Ciutadella de Menorca on the easternmost Balearic island of Menorca (Spain). Their external morphology suggested that both specimens belonged to the invasive blue planarian *Caenoplana coerulea*, a species which is native to eastern Australia. Sequence data of a fragment of the mitochondrial cytochrome *c* oxidase subunit I (COI) and of the entire 18S ribosomal RNA confirm its identification. This is one of the first records of the species in Europe where it has only been found in one locality in the United Kingdom, France and NE Spain.

## Introduction

Several species of terrestrial planarian are known as invasive, exotic species in soils of the northern hemisphere. For instance, in North America and the British Isles about a dozen species of exotic terrestrial planarians have been introduced (Jones 1988; Jones and Boag 1996; Ogren and Kawakatsu 1998). Many of these introduced exotic species are predators of earthworms, isopods and snails (e.g. Ogren 1995; Fiore et al. 2004; Sugiura et al. 2006; Iwai et al. 2010; Sugiura 2010). As such, these flatworms may pose a threat to local biodiversity (Santoro and Jones 2001). Because of this, and in view of their rapid dispersal as well as their wide distribution, these introduced exotic terrestrial flatworms are of serious agricultural and nature conservation concern.

The impacts of introduced exotic terrestrial flatworms may be especially detrimental in islands and archipelagos that support an endemic invertebrate fauna. This is illustrated by the terrestrial flatworm *Platydemus manokwari* De Beauchamp, 1962, which has been introduced in many Pacific islands (e.g. Eldredge and Smith 1995) and is considered a cause of the rapid decline of endemic land snails on these islands (Chiba 2003; Okochi et al. 2004; Ohbayashi et al. 2005; Sugiura et al. 2006; Sugiura 2009; Sugiura and Yamaura 2010). Therefore the species is of serious concern in the conservation of the unique land snail fauna of archipelagos and therefore has been included in the list of the world’s 100 worst invasive alien species (see http://www.issg.org/worst100_species.html, Lowe et al. 2000). Hence, in order to develop strategies to reduce further spread and to control their impacts on local invertebrates, rapid and accurate identifications of exotic terrestrial flatworms are essential.

Against this background, we here report for the first time the occurrence of the invasive blue land planarian *Caenoplana coerulea* Moseley, 1877 in the Balearic Islands (Menorca, Spain). Its identification was confirmed by DNA sequence analysis of the entire nuclear 18S ribosomal RNA (18S rDNA) gene and of a portion of the mitochondrial cytochrome *c* oxidase subunit 1 (COI) gene.

## Materials and methods

**Figure 1.**
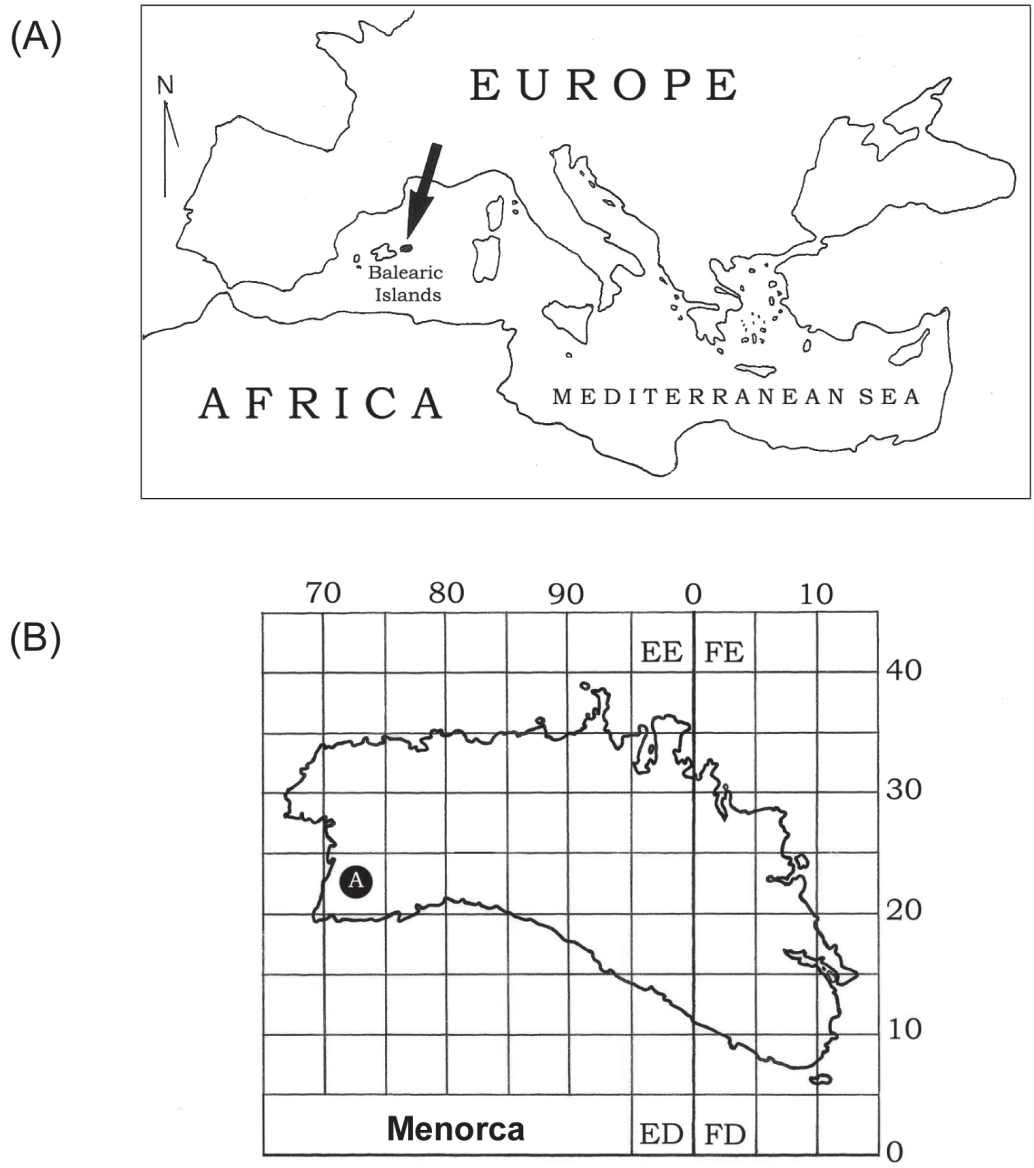
(**A**) Location of the Balearic Islands in the Mediterranean Sea. Menorca is in black and indicated by an arrow. (**B**) Detailed map of Menorca: the locality where Caenoplana coerulea was found is indicated with the letter A.

**Figure 2.**
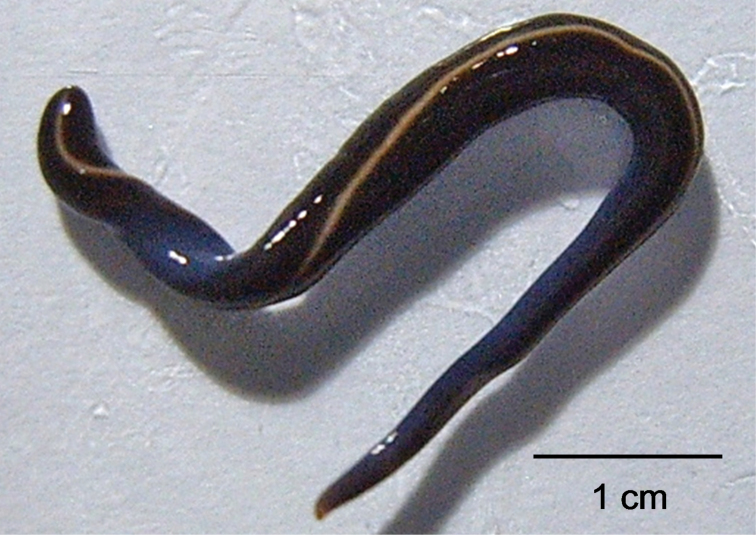
One of the two specimens of *Caenoplana coerulea* collected on Menoca.

In April 2009 two specimens of a terrestrial flatworm were collected by hand under a rock in an orchard at Ciutadella de Menorca on the easternmost Balearic island of Menorca (Spain, 39°57'00"N, 03°51'00"E; Figures 1 and 2). Both specimens (labelled ‘1957’ and ‘1958’) were stored in 100% ethanol.

Genomic DNA was extracted using the NucleoSpin® Tissue Kit (Machery-Nagel, Düren, Germany). A 424 bp fragment of the COI gene was amplified using the primer pair flatCOIL and flatCOIH (modified from Bessho et al. 1997; Table 1). PCR was performed in a total volume of 25 µl, containing 2 µl of DNA and 0.2 µM of each primer, and using the Qiagen® Multiplex PCR Kit with HotStarTaq® DNA polymerase and a final concentration of 3 mM MgCl_2_. The PCR profile was 15 min at 95 °C followed by 35 cycles of 45 s at 95 °C, 45 s at 50 °C and 1 min at 72 °C, and with a final extension step of 10 min at 72 °C. The entire 18S rDNA gene was amplified using the primer pair 4F18S and 16R18S (Winnepenninckx et al. 1994, Table 1). PCR was performed in a total volume of 25 µl containing 2 µl of DNA, 0.2 µM of each primer, 200 µM of each dNTP, 0.62 units of Taq DNA polymerase (Qiagen) and mQ-H_2_O. Triclad flatworms are known to have two types of 18S rDNA genes (Type I and II) (Carranza et al. 1996, 1999). Therefore, 18S rDNA PCR products were cloned using TOPO TA Cloning® Kit for Sequencing (Invitrogen) following the suppliers’ instructions. Fifteen colonies of each specimen were amplified as described above.

**Table 1.** Forward (F) and reverse (R) primers used for amplification and sequencing of the mitochondrial cytochrome *c* oxidase subunit I (COI) and the nuclear 18S ribosomal RNA (18S rDNA) genes of the two *Caenoplana* specimens in this study.

**Name**	**Sequence 5’-3’**	**Source**
COI:		
F: flatCOIL	GCAGTTTTTGGTTTTTTGGACATCC	modified from Bessho et al. (1997)
R: flatCOIH	GAGCAACAACATAATAAGTATCATG	modified from Bessho et al. (1997)
18S rDNA:		
F: 4F18s	CTGGTTGATYCTGCCAGT	Winnepenninckx et al. (1994)
R: 10R18S	TTGGYRAATGCTTTCGC	Winnepenninckx et al. (1994)
F: 9F18S	CGCGGTAATTCCAGCTCCA	Winnepenninckx et al. (1994)
R: 3R18S	GACGGGCGGTGTGTRC	Winnepenninckx et al. (1994)
F: 14F18S	ATAACAGGTCTGTGATGCCC	Winnepenninckx et al. (1994)
R: 16R18S	CYGCAGGTTCACCTACRG	Winnepenninckx et al. (1994)

All PCR products were purified using NucleoFast 96 PCR plates (Macherey-Nagel, Düren, Germany) and bidirectionally sequenced using the BigDye Terminator v1.1 chemistry on an ABI 3130xl automated capillary DNA sequencer (Life Technologies). For the sequencing of 18S rDNA several internal primers were used (Table 1). Sequences were visually inspected and aligned in SeqScape v2.5 (Life Technologies). COI and 18S rDNA sequences from other flatworm species of the Continenticola (see e.g. Álvarez-Presas et al. 2008, Sluys et al. 2009) were imported from GenBank (See Appendix). Sequence data sets were aligned in MAFFT v6.861 (Katoh and Toh 2008) and trimmed at 296 bp for the COI and at 1765 bp for the 18S rDNA fragment. From the Menorca specimens only 18S rDNA clones that yielded sequences without ambiguous positions were retained for further analyses.

Two tree reconstruction methods were implemented: Neighbor-Joining (NJ) (Saitou and Nei 1987) and Maximum Likelihood (ML). The most appropriate nucleotide substitution models for ML were selected using JMODELTEST v0.1.1 (Posada 2008). These were the GTR+G model for the COI fragment and the GTR+I+G model for the 18S rDNA fragment. NJ trees were made in MEGA v5.0 (Tamura et al. 2007) using K2P distances and with complete deletion of indels. ML trees were made in PAUP* v4.0b10 (Swofford 2002) using a heuristic search with the tree-bisection-reconnection branch-swapping algorithm and random addition of taxa. Trees were rooted with *Bdelloura candida* (Girard, 1850) (Maricola, family Bdellouridae). Branch support was assessed via nonparametric bootstrapping using 1000 bootstrap replicates for NJ or 200 bootstrap replicates for ML (Felsenstein 1985). Only nodes with bootstrap values of ≥ 70% were retained and considered meaningful (Hillis and Bull 1993). P-distances were calculated with MEGA v5.0.

Both specimens have been deposited in the collections of the Royal Belgian Institute of Natural Sciences, Brussels, under catalogue number IG.32062. DNA sequences have been deposited in GenBank under accession numbers JQ639215-JQ639227 (for 18S rDNA) and JQ514564 (for COI).

## Results and discussion

**Figure 3.**
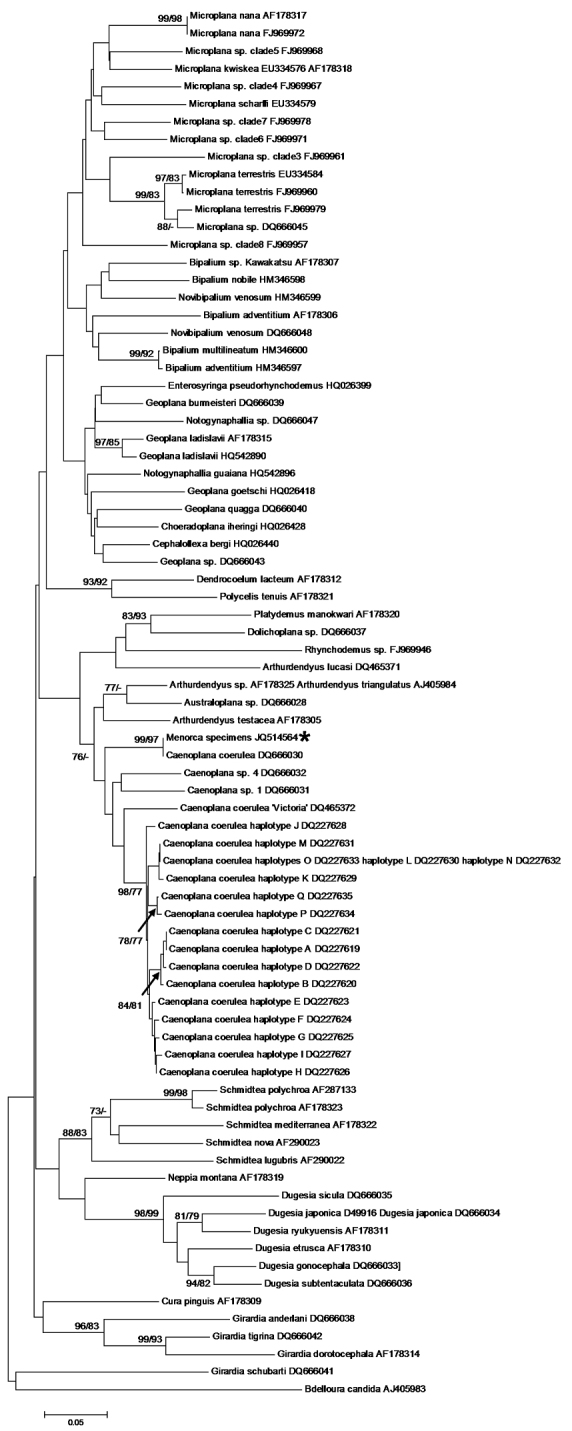
Neighbor-Joining and ML tree of the 296 bp dataset of the mitochondrial cytochrome *c* oxidase subunit I gene (COI). The haplotype of the Menorcan specimens is indicated with an asterisk. Bootstrap values ≥ 70% for the NJ and ML trees are given as NJ/ML and are shown at the nodes. – indicates that the node was not supported by the analysis.

**Figure 4.**
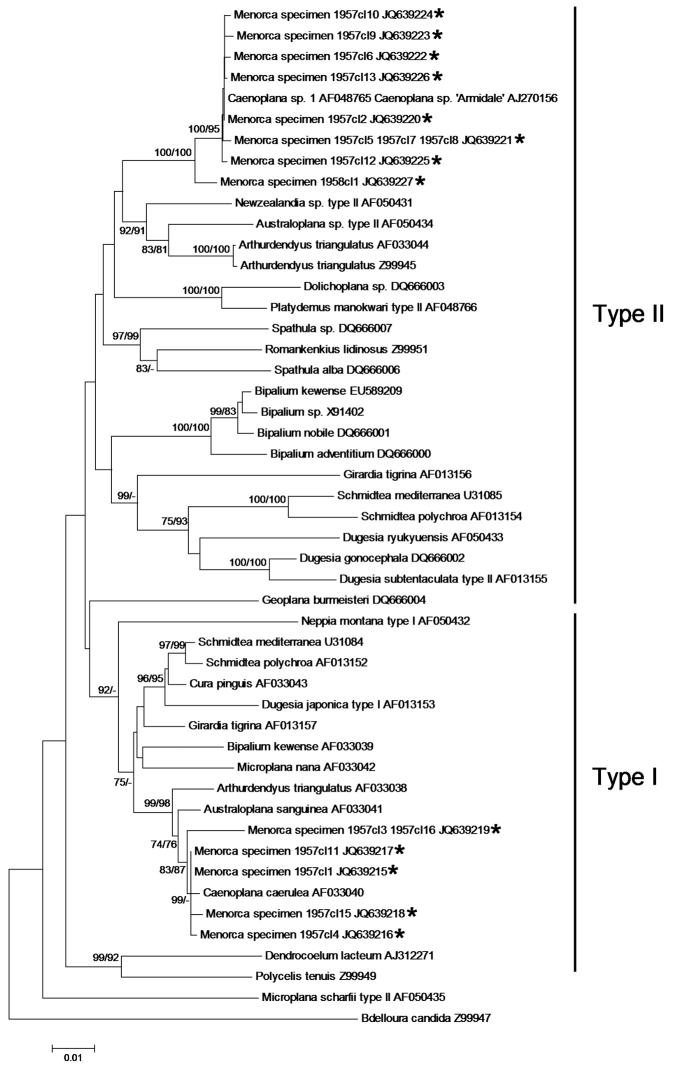
Neighbor-Joining and ML tree of the 1765 bp dataset of the nuclear 18S rDNA gene. The clones (cl) of the Menorcan specimens ‘1957’ and ‘1958’ are indicated with an asterisk. Bootstrap values ≥ 70% for the NJ and ML trees are given as NJ/ML and are shown at the nodes. – indicates that the node was not supported by the analysis. Note that the clades of the type I and type II 18S rRNA variants are not supported.

The dorsal dark blue ground-colour with a thin median dorsal stripe, the intense blue colour of the ventral side, and eyes that are arranged in a single row around the anterior tip and which do not extend dorsally, suggest that the two specimens belong to the species of blue land planarian, *Caenoplana coerulea* Moseley, 1877 (Geoplanidae). This is corroborated by our phylogenetic analysis of the COI and 18S rDNA genes. Both individuals had the same COI haplotype; as in other triclads, there were two different intra-individual types of 18S rDNA (Carranza et al. 1996, 1999). We found five type I and eight type II 18S rDNA variants. Figures 3–4 show the phylogenetic trees inferred from the COI and 18S rDNA data, respectively. The COI haplotype of the Menorcan specimens clustered with strong support with a haplotype of *Caenoplana coerulea* from the UK (GenBank accession number DQ666030), from which it differed by only one, ambiguous position (i.e. a G for DQ666030, while ‘N’ for the Menorcan haplotype). The mean P-distance between the COI haplotype from Menorca and the other *Caenoplana coerulea* haplotypes was 0.10 ± 0.02, whereas the P-distance with other Geoplanid species was higher (0.16 ± 0.03) and comparable to what we found among Geoplanidae taxa (0.17 ± 0.03). The 18S rDNA type I sequences from the Menorcan specimens formed a strongly supported clade with *Caenoplana coerulea* AF033040 (from the UK) (mean P-distance: 0.008 ± 0.002), whereas those of 18S rDNA type II formed a strongly supported clade with *Caenoplana* sp.1 AF048765 (unknown origin) and *Caenoplana* sp. ‘Armidale’ AJ270156 (from Australia) (mean P-distance: 0.003 ± 0.001). The mean P-distance between the Menorcan type I and type II sequences and sequences from the other geoplanid species was substantially higher, viz. 0.019 ± 0.003 and 0.058 ± 0.005, respectively.

*Caenoplana coerulea* is native to eastern Australia but, as a result of human activities, it has been introduced to New Zealand, the United States, the United Kingdom, Norfolk Island (Australia), and France (Ogren 1989; Winsor 1998; Jones 1998, 2005), and more recently in Argentina (Luis-Negrete et al. 2011) and NE Spain (Mateos et al. 2012). After introduction, the species may expand its range rapidly. For example, since its accidental introduction into the USA prior to 1943, it has spread rapidly over a large part of the country (California: 1943, Florida: 1961, Georgia: 1972, Texas: 1978, Iowa: 1999, North Carolina: 2001) (Ogren 2001). Whether this fast expansion is due to its high intrinsic dispersal capacity or due to repeated, independent introductions, is unknown.

In the Iberian Peninsula and Balearic Islands, at present ten autochthonous species of the family Geoplanidae have been reported (Mateos et al. 1998, 2009; Vila-Farré et al. 2008, 2011). In addition, two introduced species, *Bipalium kewense* Moseley, 1878 (Bipaliidae; recorded from Barcelona) (Filella-Subirá 1983) and *Platydemus* sp. (Geoplanidae; recorded from Benamargosa, Málaga) (Vila-Farré et al. 2011), have been reported from the Iberian Peninsula but not from the Balearic Islands where only *Microplana terrestris* (O.F. Müller, 1774) (Geoplanidae) has been found (Minelli 1977). Hence, the present record of two specimens of *Caenoplana coerulea* implies the first introduced species of Geoplanidae in the Balearic Islands. Very recently, the species was also found on the Iberian Peninsula (La Garrotxa, Girona province) (Mateos et al. 2012). Also, pictures of the species that were taken in Spain (Boadilla del Monte, October 2010 and Girona, 22 December 2011) can be found at http://www.flickr.com/photos/51708886@N03/6351086047/ and http://www.biodiversidadvirtual.org/insectarium/Caenoplana-coerulea-img293381.html, respectively. In Europe, the species is further only known from a hothouse in Liverpool (Jones 1998, 2005) and one locality in France (Ogren 1989; Winsor 1998; Winsor et al. 2004).

We do not know when exactly this exotic species arrived in the Balearic Islands. The first specimens of *Caenoplana coerulea* were found in an orchard in April 2009. In 2011 the species had spread to a nearby garden, where it was found at shaded places. As is the case in other land planarians, its spread and distribution in newly colonized areas is probably mainly determined by moisture (Fraser and Boag 1998). Even in its native region (Australia), the distribution of *Caenoplana coerulea* is restricted to areas with a high humidity (Luis-Negrete et al. 2011). Even though the impact of *Caenoplana coerulea* on earthworm and terrestrial gastropod populations is not known, the species is at least reported to feed on isopods, diplopods, earwigs, and snails (Olewine 1972; Barnwell 1978; Terrace and Baker 1994; Jones 2005). Its broad food spectrum might facilitate the establishment and possible spread of the species in Spain and, eventually, elsewhere in Europe.

## Figures and Tables

**Table:** List of samples used in this study with GenBank accession numbers and sampling locality (if known). The classification follows Sluys et al. (2009).

**Species**	**18S rDNA**		**COI**	**Sampling locality**
	**Type I**	**Type II**		
**Maricola**				
**Family Bdellouridae**				
Subfamily Bdellourinae				
*Bdelloura candida*	Z99947		AJ405983	
**Continenticola**				
**Family Planariidae**				
*Polycelis tenuis*	Z99949		AF178321	Spain
**Family Dendrocoelidae**				
*Dendrocoelum lacteum*	AJ312271		AF178312	France
**Family Dugesiidae**				
*Cura pinguis*	AF033043		AF178309	New Zealand
*Dugesia etrusca*			AF178310	Italy
*Dugesia gonocephala*		DQ666002	DQ666033	The Netherlands
*Dugesia japonica*	AF013153	D83382	DQ666034	Japan
	D49916			
*Dugesia ryukyuensis*		AF050433	AF178311	Japan
*Dugesia sicula*			DQ666035	Spain
*Dugesia subtentaculata*		AF013155	DQ666036	Spain
*Girardia anderlani*		DQ666013	DQ666038	Brasil
*Girardia dorotocephala*			AF178314	USA
*Girardia schubarti*		DQ666015	DQ666041	Brasil
*Girardia tigrina*	AF013157	AF013156	DQ666042	France
*Neppia montana*	AF050432		AF178319	
*Romankenkius libidinosus*		Z99951		
*Schmidtea mediterranea*	U31084	U31085	AF178322	Spain
*Schmidtea lugubris*			AF290022	
*Schmidtea nova*			AF290023	
*Schmidtea polychroa*	AF013152	AF0131154	AF178323	Spain
	AF287133			
*Spathula alba*		DQ666006		New Zealand
*Spathula* sp.		DQ666007		New Zealand
**Family Geoplanidae**				
**Subfamily Bipaliinae**				
*Bipalium adventitium*		DQ666000	AF178306	USA
			HM346597	
*Bipalium kewense*	AF033039			
		EU589209		Japan
*Bipalium multilineatum*			HM346600	Japan / South Korea
*Bipalium nobile*	DQ666001			Japan
	HM346598			
*Bipalium* sp. ‘Kawakatsu’		X91402	AF178307	Japan
*Novibipalium venosum*			DQ666048	Japan
			HM346599	South Korea
**Subfamily Microplaninae**				
*Microplana kwiskea*			EU334576	Spain
			AF178318	
*Microplana nana*	AF033042		AF178317	Spain
			FJ969972	Spain
*Microplana scharffi*		AF050435	EU334579	UK
*Microplana terrestris*			EU334584	
			FJ969960	Spain
			FJ969979	Spain
*Microplana* sp.			DQ666045	Spain
*Microplana* sp. clade 3			FJ969961	Spain
*Micorplana* sp. clade 4			FJ969967	Spain
*Microplana* sp. clade 5			FJ969968	Spain
*Microplana* sp. clade 6			FJ969971	Spain
*Microplana* sp. clade 7			FJ969978	Spain
*Microplana* sp. clade 8			FJ969957	Spain
**Subfamily Rhynchodeminae**				
*Arthurdendyus lucasi*			DQ465371	
*Arthurdendyus testacea*			AF178305	Australia
*Arthurdendyus* sp.			AF178325	Australia
*Arthurdendyus triangulatus*	AF033038	AF033044	AJ405984	
		Z99945		
*Australoplana sanguinea*	AF033041			Australia
*Australoplana* sp.		AF050434	DQ666028	Australia
*Caenoplana coerulea*	AF033040		DQ666030	UK
‘Victoria’			DQ465372	Australia
haplotype A			DQ227619	Australia
haplotype B			DQ227620	Australia
haplotype C			DQ227621	Australia
haplotype D			DQ227622	
haplotype E			DQ227623	Australia
haplotype F			DQ227624	Australia
haplotype G			DQ227625	Australia
haplotype H			DQ227626	Australia
haplotype I			DQ227627	
haplotype J			DQ227628	Australia
haplotype K			DQ227629	Australia
haplotype L			DQ227630	Australia
haplotype M			DQ227631	Australia
haplotype N			DQ227632	Australia
haplotype O			DQ227633	Australia
haplotype P			DQ227634	Australia
haplotype Q			DQ227635	Australia
1957			JQ514564	Spain (Menorca)
1958			JQ514564	Spain (Menorca)
1957clone1	JQ639215			Spain (Menorca)
1957clone4	JQ639216			Spain (Menorca)
1957clone11	JQ639217			Spain (Menorca)
1957clone15	JQ639218			Spain (Menorca)
1957clone3-16	JQ639219			Spain (Menorca)
1957clone2		JQ639220		Spain (Menorca)
1957clone5-7-8		JQ639221		Spain (Menorca)
1957clone6		JQ639222		Spain (Menorca)
1957clone9		JQ639223		Spain (Menorca)
1957clone10		JQ639224		Spain (Menorca)
1957clone12		JQ639225		Spain (Menorca)
1957clone13		JQ639226		Spain (Menorca)
1958clone1		JQ639227		Spain (Menorca)
*Caenoplana* sp.’Armidale’		AJ270156		Australia
*Caenoplana* sp. 1		AF048765	DQ666031	
*Caenoplana* sp. 4			DQ666032	
*Dolichoplana* sp.		DQ666003	DQ666037	
*Newzealandia* sp.		AF050431		
*Platydemus manokwari*		AF048766	AF178320	Australia
*Rhynchodemus* sp.			FJ969946	
**Subfamily Geoplaninae**				
*Cephaloflexa bergi*			HQ026440	
*Choeradoplana iheringi*			HQ026428	Brasil
*Enterosyringa pseudorhynchodemus*		HQ026399		
*Geoplana burmeisteri*		DQ666004	DQ666039	Brasil
*Geoplana goetschi*			HQ026418	
*Geoplana ladislavii*			AF178315	Brasil
			HQ542890	
*Geoplana quagga*			DQ666040	Brasil
*Geoplana* sp.			DQ666043	Uruguay
*Notogynaphallia guaiana*			HQ542896	
*Notogynaphallia* sp.			DQ666047	Brasil
